# Elderly Peritoneal Dialysis Compared with Elderly Hemodialysis Patients and Younger Peritoneal Dialysis Patients: Competing Risk Analysis of a Korean Prospective Cohort Study

**DOI:** 10.1371/journal.pone.0131393

**Published:** 2015-06-29

**Authors:** Hyunsuk Kim, Jung Nam An, Dong Ki Kim, Myoung-Hee Kim, Ho Kim, Yong-Lim Kim, Ki Soo Park, Yun Kyu Oh, Chun Soo Lim, Yon Su Kim, Jung Pyo Lee, CRC for ESRD Investigators

**Affiliations:** 1 Department of Internal Medicine, Seoul National University College of Medicine, Seoul, Korea; 2 Department of Internal Medicine, Seoul National University Boramae Medical Center, Seoul, Korea; 3 Clinical Research Center for End Stage Renal Disease in Korea, Daegu, Korea; 4 Department of Dental Hygiene, College of Health Science, Eulji University, Seongnam, Korea; 5 Department of Biostatistics and Epidemiology, Graduate School of Public Health & Asian Institute for Energy, Environment and Sustainability, Seoul National University, Seoul, Korea; 6 Department of Internal Medicine, Kyungpook National University School of Medicine, Daegu, Korea; 7 Department of Preventive Medicine and Institute of Health Sciences, Gyeongsang National University School of Medicine, Jinju, Korea; Sao Paulo State University, BRAZIL

## Abstract

The outcomes of peritoneal dialysis (PD) in elderly patients have not been thoroughly investigated. We aimed to investigate the clinical outcomes and risk factors associated with PD in elderly patients. We conducted a prospective observational nationwide adult end-stage renal disease (ESRD) cohort study in Korea from August 2008 to March 2013. Among incident patients (n = 830), patient and technical survival rate, quality of life, and Beck’s Depression Inventory (BDI) scores of elderly PD patients (≥65 years, n = 95) were compared with those of PD patients aged ≤49 years (n = 205) and 50~64 years (n = 192); and elderly hemodialysis (HD) patients (n = 315). The patient death and technical failure were analyzed by cumulative incidence function. Competing risk regressions were used to assess the risk factors for survival. The patient survival rate of elderly PD patients was inferior to that of younger PD patients (P<0.001). However, the technical survival rate was similar (P = 0.097). Compared with elderly HD patients, the patient survival rate did not differ according to dialysis modality (P = 0.987). Elderly PD patients showed significant improvement in the BDI scores, as compared with the PD patients aged ≤49 years (P = 0.003). Low albumin, diabetes and low residual renal function were significant risk factors for the PD patient survival; and peritonitis was a significant risk factor for technical survival. Furthermore, low albumin and hospitalization were significant risk factors of patient survival among the elderly. The overall outcomes were similar between elderly PD and HD patients. PD showed the benefit in BDI and quality of life in the elderly. Additionally, the technical survival rate of elderly PD patients was similar to that of younger PD patients. Taken together, PD may be a comparable modality for elderly ESRD patients.

## Introduction

In recent decades, the prevalence and the incidence of elderly patients undergoing renal replacement therapy (RRT) have been continuously increasing [1]. The management in elderly patients with end-stage renal disease (ESRD) includes RRT such as kidney transplantation [2, 3], dialysis, as well as maximal conservative management [3–5]. The selection of management is not easy because of the simultaneous benefit and burden of each modality [6]. Moreover, choosing modality in the elderly is more difficult because clinicians have to base their choice between the complexity of co-morbidity [7] and cost or quality of life (QOL) [8]. In fact, for dialysis candidates with ESRD, it is critical to determine whether peritoneal dialysis (PD) or hemodialysis (HD) is more effective, as it may directly affect the survival rate and QOL of these patients [9].

Recently, PD has been less frequently utilized [10], owing not only to the autonomy, comorbidity, and performance of the patients, but also financial, resource availability, and cultural issues [11]. Moreover, in Korea, incident PD patients are decreasing, as compared to HD patients [12, 13].

The outcomes and risk factors of PD in elderly patients are controversial [14]. Studies described that there was no difference between PD and HD for elderly patients in terms of the mortality [15, 16]; whereas another study reported that the mortality rate of PD patients was higher than that of HD patients [17, 18]. Furthermore, one study showed that older PD patients showed inferior survival than younger PD patients [11], while another found no difference in overall survival when comparing PD patients according to age [19]. Additionally, Kurella M et al. reported that 1-year survival for octogenarians and nonagenarians on dialysis was not different [20].

Moreover, the preferences of each patient are important for selecting the appropriate modality. One report indicated that more than one-third of elderly patients without contraindication preferred PD over HD [21]. Similarly, another study from Hong Kong showed that 75% of elderly patients preferred PD [22]. Accordingly, helping elderly patients decide whether they wish to receive home dialysis is an important role of medical professionals.

To date, there are limited prospective studies about the effects of PD on the clinical outcomes in elderly patients, especially in Asian populations [23]. Thus, the purpose of this study was to investigate the patient and technical survival rates and risk factors of survival in a prospective Korean ESRD cohort.

## Materials and Methods

### Study Participants

**Fig 1:**
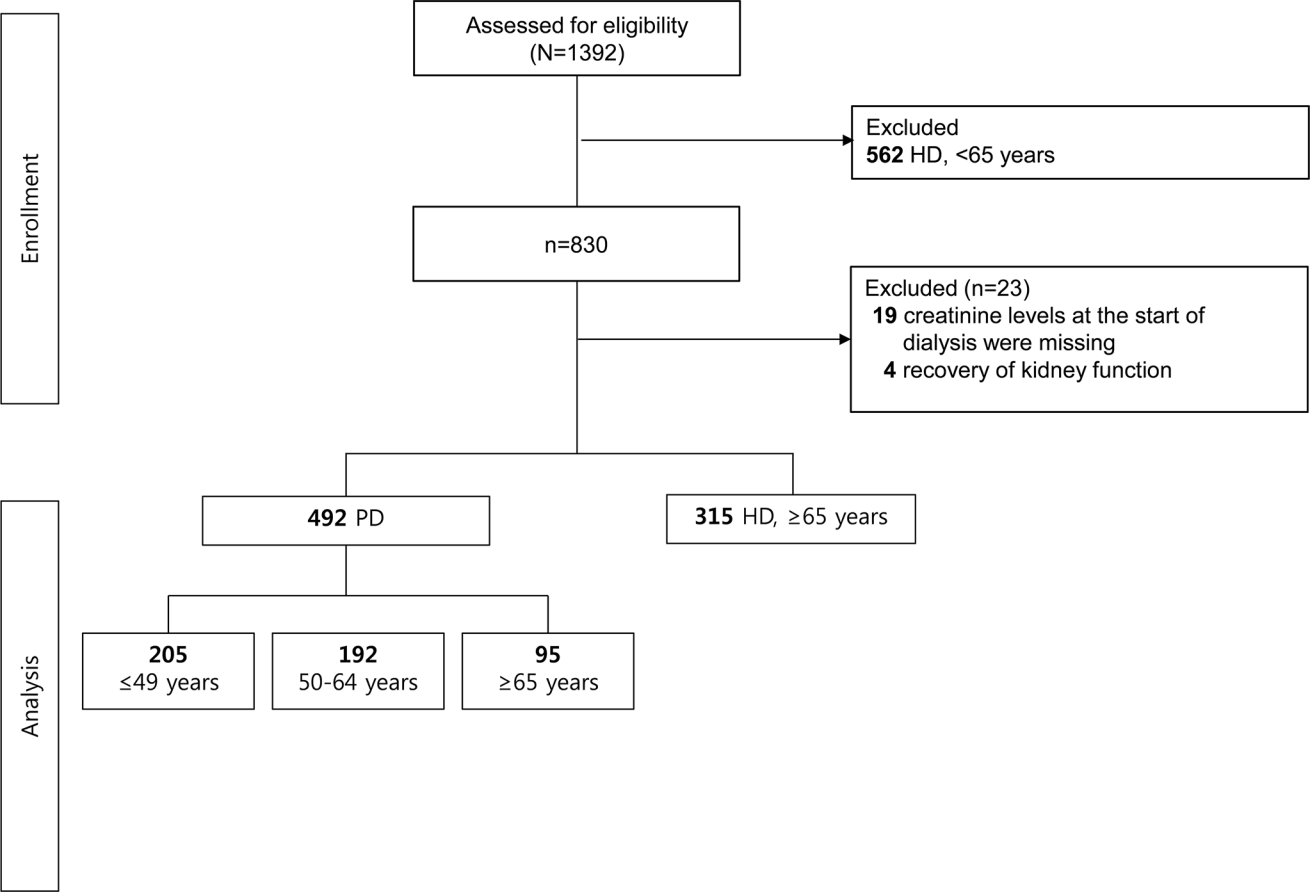
Study flow. 492 patients undergoing PD were enrolled and divided into 3 groups according to age (≤49 years, n = 205; 50~64 years, n = 192; ≥65 years, n = 95); 315 HD patients aged ≥65 years were enrolled for comparison with elderly PD patients.

The Clinical Research Center for End Stage Renal Disease (CRC for ESRD) cohort is a nationwide, multi-center, web-based, prospective cohort of chronic kidney disease (CKD) patients undergoing dialysis in South Korea [24–25]. The CRC for ESRD cohort began to register ESRD patients on dialysis in July 2008, and 31 hospitals in South Korea are currently participating. Patients who were at least 20 years old and began treatment with maintenance dialysis due to ESRD between July 2008 and March 2013 were eligible for the study. Elderly patients were defined based on ROC curve of age and patient death; our data indicated that an age 63.5 or 64.5 years is the best cut-off value (ROAUC: 0.695 [P<0.001]; 63.5 year: sensitivity 0.821, specificity 0.480; 64.5 year: sensitivity 0.808, specificity 0.488). We therefore set the cut-off at 65 years to differentiate elderly patients. Among adult patients (≥20 years old, n = 830) initiated on maintenance dialysis, 492 patients undergoing PD were enrolled and divided into 3 groups according to age (≤49 years, n = 205, 50~64 years, n = 192; ≥65 years, n = 95). In addition, 315 HD patients aged ≥65 years were enrolled for comparison with elderly PD patients. Patients who stopped dialysis due to recovery of kidney function (n = 4) or whose creatinine levels at the start of dialysis (n = 19) were missing were excluded. Finally, 807 patients were included in the analysis (Fig 1). All patients participated voluntarily after providing written informed consent. The study was approved by the Institutional Review Board at each center and conducted in accordance with the Declaration of Helsinki.

Data were extracted from the CRC for ESRD web database (http://webdb.crc-esrd.co.kr): for outcome analysis. Baseline information at enrollment included age, sex, height, weight, primary renal disease, modified Charlson co-morbidity index (mCCI), Karnofsky performance status (KPS), subjective global assessment (SGA), laboratory data, and dialysis information (PD [assisted PD or continuous ambulatory PD] and HD [from 1 to 5 times a week]). Comorbidities, laboratory data, 24-h urine volume and dialysis information were followed at 3 and 6 months after the start of dialysis and at 6-month intervals thereafter. Laboratory data and 24-h urine volume were analyzed using time-averaged values.

KPS, SGA data was followed with intervals of 12 months. The estimated glomerular filtration rate (eGFR) was calculated using CKD-Epidemiology Collaboration equations just before the RRT [26]. The mCCI was calculated for each patient at the initiation of dialysis to predict 1-year mortality [27, 28]. Dialysis modality was defined as the modality 3 months after the first dialysis, or the modality at dialysis initiation if death occurred before 3 months. Familial and social support were defined according to the degree of assistance from each patient’s family or society at the time of data entry and was indicated by a subjective percentage value consisting of 4 levels.

### Clinical Outcomes

The primary outcome was the patient and technical survival rate after starting dialysis. The secondary outcomes were the reasons for patient death and technical failure, incidence and microbiology of peritonitis, the 1-year changes in Kidney Disease Quality of Life-36 (KDQOL-36) and BDI scores. Technical failure included alteration from HD to PD or vice versa. Hospitalization was defined as admission for ≥24 hours, except for diagnostic work-ups for transplantation. Cardiovascular events included clinical events requiring admission, such as ischemic heart disease, congestive heart failure, arrhythmia, and cerebrovascular disease. RRT-associated complications included vascular events requiring angioplasty, surgical intervention, or changes in vascular catheters for HD; and peritonitis or catheter malposition or malfunction in PD. Peritonitis was defined as the presence of: 1) signs and symptoms of peritoneal inflammation, and 2) a peritoneal effluent white blood cell count of >100 cells/mm^3^ and >50% neutrophils [29].

### Survey Instruments

KDQOL-36 is used to evaluate the health-related QOL of ESRD patients [30]. We utilized the Korean version [31]; this includes 12 generic chronic disease items (the short form [SF]-12) and 24 additional items (kidney-disease-targeted items: symptom/problem list, 12 items; effects of kidney disease, 8 items; and burden of disease, 4 items). The item scores were aggregated without weighting and transformed linearly to a 0–100 range, with higher scores indicating better states.

The Korean version of BDI was used to evaluate depression [32]. The BDI consisted of 21 self-reported items, and each item was rated between 0–3, producing a possible score range of 0–63, with higher scores indicating more severe depression.

The KPS was used to assess the subjects’ performance status, and was defined as follows: KPS score ≥80: able to carry out normal activity and work, no special care needed; 70–50: unable to work, able to live at home and care for most personal needs, varying amount of assistance needed; KPS ≤40: unable to care for self, requires equivalent of institutional or hospital care, disease may be progressing rapidly.

For nutritional status evaluation, SGA scores were divided into 3 categories (category 1: well-nourished [SGA score, 6–7]; 2: mild-to-moderately malnourished [3–5]; and 3: severely malnourished [1–2]). Because the number of subjects classified as category 3 was small, we classified the 3 SGA categories into 2 groups (category 1 *vs*. 2 and 3).

### Statistical Analysis

Continuous variables were expressed as mean and standard deviation, and categorical variables were presented as frequencies with percentages. For comparison of patient survival and technical survival rate, considering the low frequency of each outcome, patients undergoing PD were divided into younger and elderly groups based on the cut-off age (65 years) for analysis. Continuous variables were analyzed using Student’s t-test or Wilcoxon rank sum test depending on whether the data were normally distributed. Categorical variables were compared between the groups using Chi-square test or Fisher’s exact test. Transplantation and technical survival were regarded as competing risk events in the survival of PD patients, and technical survival was only considered a competing risk event in the survival of elderly patients because there was no transplantation as a competing event in elderly patients. Patient death and transplantation were regarded as competing risk events in the technical survival of PD patients. The cumulative incidence function was compared between groups using Gray’s method and was shown on a plot [33]. Differences in mortality rates or technical failure rates were compared using a Fine and Gray model (competing risks regression) [34]. Univariate analysis using Competing Risks Regression was performed to determine the risk factors for patient or technical survival, followed by stepwise multivariate analysis for determining significant factors based on a significance level of 0.2. After confirming the interaction of each significant variable, the final model included only those factors with a significance level of 0.05. The linearity assumption of continuous variables was verified. Continuous variables such as laboratory findings were categorized according to tertile and proportional hazards assumption of categorical variables was verified using log-minus-log plot. For comparisons of QOL and BDI, Student’s t-test and repeated measure ANOVA were used. IBM SPSS software (version 21.0; Armonk, NY, USA) and R statistical software (R Foundation for Statistical Computing, Vienna, Austria, http://www.R-project.org/) were used for all analyses. A 2-tailed P-value <0.05 was considered significant.

## Results

### Patient Characteristics

The patient demographics were summarized in Table 1. PD patients were divided to 3 groups according to age; the proportion of men according to age groups was not different (P = 0.271). The ≤49-year group had less diabetic kidney disease (≤49 *vs*. 50~64 *vs*. ≥65 years: 32% *vs*. 55% *vs*. 54%, P<0.001) and more glomerulonephritis, as compared with the other age groups (≤49 *vs*. 50~64 *vs*. ≥65 years: 31% *vs*. 13% *vs*. 10%, P<0.001). The incidences of cardiovascular diseases and heart failure were higher in elderly PD patients (P<0.001), and they showed higher mCCI (8–15), lower KPS (<70), and poorer SGA (1–5) scores. Moreover, elderly PD subjects were more dependent on familial support (P = 0.002). In the laboratory findings, serum phosphorus was significantly lower and serum calcium was significantly higher in elderly than younger PD patients.

**Table 1:** Patient characteristics: age groups among peritoneal dialysis patients and peritoneal dialysis or hemodialysis groups in elderly patients.

	PD, ≤49 y	PD, 50~64 y	PD, ≥65 y	P ^a^	HD, ≥65 y	PD, ≥65 y	P ^b^
Patients (n)	205	192	95		315	95	
Age at the time of dialysis	39.0±7.5	56.1±4.3	70.3±4.4	<0.001	72.2±5.4	70.3±4.4	0.001
Gender (male), n (%)	191 (61)	122 (64)	63 (66)	0.271	191 (61)	63 (66)	0.190
Primary kidney disease, n (%)				0.539			0.578
Diabetes	65 (32)	105 (55)	51 (54)	<0.001	170 (55)	51 (54)	0.961
Hypertension	37 (18)	30 (16)	20 (21)	0.078	65 (21)	20 (21)	0.930
Glomerulonephritis	62 (31)	25 (13)	9 (10)	<0.001	21 (7)	9 (10)	0.421
Others	39 (19)	31 (17)	15 (15)	0.097	53 (24)	15 (15)	0.255
Systolic BP, mmHg, mean ± SD	136±23	137±24	138±21	0.510	143±23	138±21	0.059
Diastolic BP, mmHg, mean ± SD	81±17	79±14	76±12	0.045	73±12	76±12	0.037
BMI, kg/m^2^, mean ± SD	23±4	23±3	22±3	0.077	23±3.4	22±3	0.986
24-h urine volume, mL	920±740	1011±791	911±682	0.511	662±585	911±682	0.002
Cardiovascular comorbidity, n (%)							
Cardiovascular disease	20 (10)	44 (23)	40 (42)	<0.001	127 (40)	40 (42)	0.715
Heart failure	18 (9)	13 (7)	24 (25)	<0.001	37 (12)	24 (25)	0.001
mCCI, n (%)				<0.001			0.755
0–5	190(93)	110(58)	28(29)		75(24)	28(29)	
6–7	11(5)	60(32)	35(37)		134(43)	35(37)	
8–15	3(2)	20(10)	32(34)		102(33)	32(34)	
KPS, n (%)				<0.001			0.072
80–100	90(83)	84(76)	29(58)		98(56)	29(58)	
-70	19(17)	27(24)	21(42)		77(44)	21(42)	
SGA score, n (%)				<0.001			0.459
6–7	138(74)	115(71)	49(56)		162(57)	49(56)	
1–5	49(26)	47(29)	38(44)		119(43)	38(44)	
Familial support, n (%)				0.002			0.618
None (independent)	42 (21)	25 (13)	6 (6)		21 (7)	6 (6)	
<50%	47 (23)	52 (30)	13 (14)		53 (17)	13 (14)	
50~99%	89 (44)	83 (50)	55 (57)		159 (51)	55 (57)	
100% (dependent)	24 (12)	28 (17)	21 (23)		78 (25)	21 (23)	
Social support, n (%)				0.115			0.645
None (independent)	52 (26)	40 (21)	18 (19)		55 (18)	18 (19)	
<50%	47 (23)	64 (34)	21 (22)		84 (27)	21 (22)	
50~99%	85 (42)	66 (35)	42 (44)		122 (39)	42 (44)	
100% (dependent)	18 (9)	18 (10)	14 (15)		50 (16)	14 (15)	
Education, n (%)				0.001			0.179
Uneducated	1 (0)	4 (2)	14 (15)		28 (9)	14 (15)	
Elementary to high school	91 (45)	136 (72)	50 (53)		216 (69)	50 (53)	
University or graduate school	100 (50)	43 (22)	17 (18)		50 (16)	17 (18)	
Unknown	13 (5)	9 (4)	4 (4)		21 (6)	4 (4)	
Laboratory findings, mean ± SD							
Hemoglobin, g/dL	9.2±1.8	9.3±1.6	9.2±1.4	0.191	9.3±4.8	9.2±1.4	0.750
Albumin, g/dL	3.5±0.8	3.4±0.7	3.3±0.6	0.112	3.3±0.7	3.3±0.6	0.465
eGFR, mL/min/1.73 m^2^	6.5±2.9	8.4±12.7	8.9±3.9	0.139	8.6±5.3	8.9±3.9	0.613
Potassium, mmol/L	4.6±1.1	4.6±1.0	4.5±0.9	0.464	4.6±1.0	4.5±0.9	0.116
Calcium, mg/dL	7.8±1.2	7.8±1.1	8.0±0.8	0.002	8.2±3.8	8.0±0.8	0.716
Phosphorus, mg/dL	5.8±2.1	5.3±1.7	4.7±1.5	<0.001	4.9±1.9	4.7±1.5	0.305

Data are presented as mean ± standard deviation or n (%).

^a^ Among the 3 PD groups.

^b^ Between the 2 elderly groups.

Abbreviations: PD, peritoneal dialysis; HD, hemodialysis; BP, blood pressure; BMI, body mass index; mCCI, modified Charlson co-morbidity index; KPS, Karnofsky Performance status; SGA, subjective global assessment; eGFR, estimated glomerular filtration rate.

Next, all patients aged ≥65 years were divided and compared according to the modality used. HD patients were older than PD patients (P = 0.001); and, except for the 24-h urine volume (P = 0.001) and diastolic blood pressure (P = 0.045), all baseline parameters were similar between the 2 groups. Heart failure was more common in the elderly PD group (P = 0.001). The mCCI, KPS, and SGA scores, the familial and social supports, and laboratory findings did not differ according to the dialysis modality.

The major causes of death included cardiovascular diseases and infection. Among 82 deaths, cardiovascular (n = 40) and infection (n = 21) related deaths were the major cause of death. There were 1 hyperkalemia, 1 hypoglycemia, 4 malignancy, 1 cachexia, 4 malignant disease, 1 treatment related accident, and 13 unspecified cause. Fourteen deaths were of unknown cause. (S1 Table).

### Patient Death and Technical Failure of Elderly PD Patients Compared to Younger PD Patients and Elderly HD Patients

**Fig 2:**
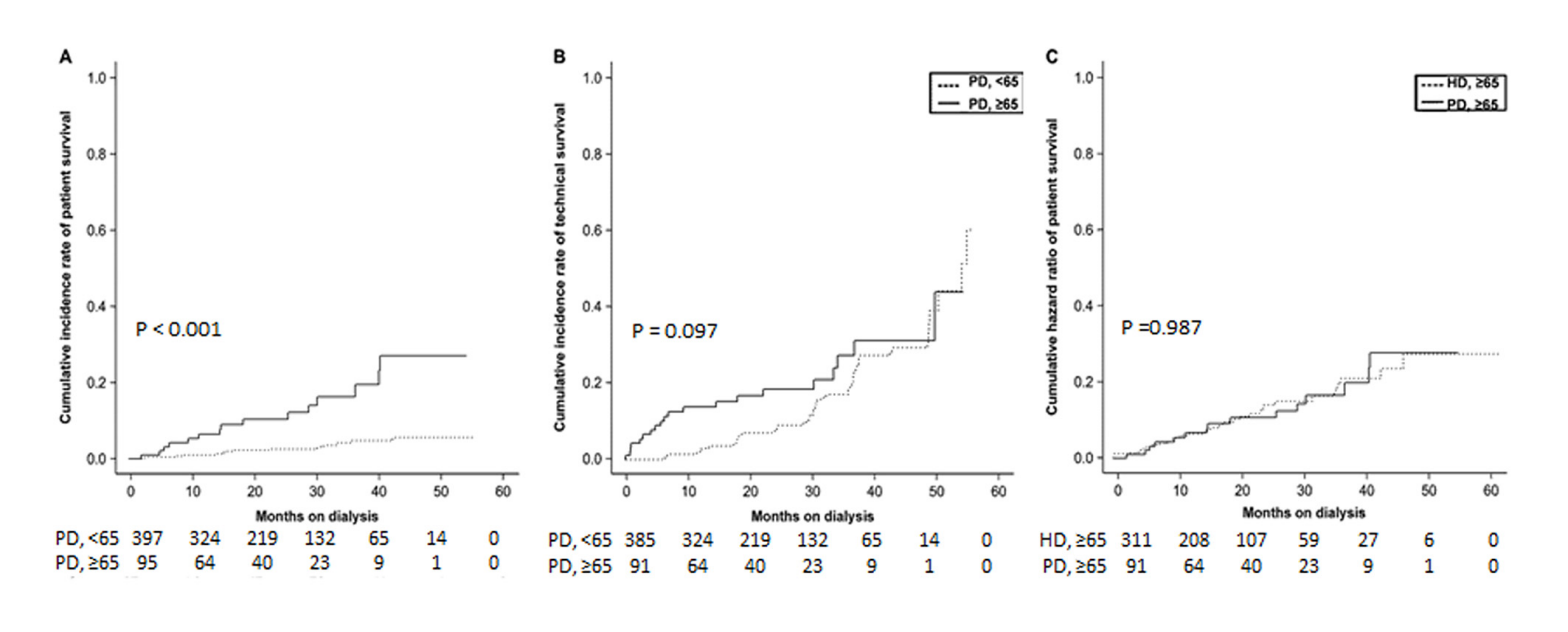
Competing risk model for patient death and technical failure of elderly (≥65 years) patients undergoing peritoneal dialysis (PD), as compared with younger PD patients and elderly hemodialysis (HD) patients. A. Comparison of patient death in younger (<65 years) patients undergoing PD. Transplantation and technical failure were considered competing risk events when examining patient death. B. Comparison of technical failure in younger (<65 years) patients undergoing PD. Patient death and transplantation were considered competing risk events when examining technical failure. C. Comparison of patient death in elderly patients undergoing HD. Transplantation was considered a competing risk event when examining patient death because there was no transplantation as a competing event in elderly patients.

In cumulative incidence function test among PD groups, the elderly PD group was associated with a significantly higher patient death rate, as compared with the younger age groups (Fig 2A, P<0.001). However, the technical failure rates were similar among the 2 PD groups (Fig 2B, P = 0.097). When age, hemoglobin, phosphorus, albumin, potassium, 24-h urine volume, SGA, and diabetes were adjusted in stepwise multivariate analysis, the risk of death among elderly PD patients was 2.99 times higher than that in the younger PD group with a statistically significant difference (P = 0.009, Table 2). In addition, after adjusting for age, albumin, 24-h urine volume, diabetes, and peritonitis, the risk of technical failure was 1.43 times higher in the elderly PD group, as compared to the younger PD group; however, the difference was not statistically significant (P = 0.220, Table 2).

**Table 2:** Independent risk factors of patient death or technical failure in peritoneal dialysis patients.

Patient death		Univariate competing risk regression		Multivariate competing risk regression*	
Variable	Category	SHR [95% CI]	P-value	SHR [95% CI]	P-value
Age	Elderly PD (vs. younger PD)	5.18 [2.52, 10.60]	<0.001	2.99 [1.32, 6.76]	0.009^a^
Sex	Female (vs. male)	0.79 [0.37, 1.70]	0.550		
Hemoglobin (g/dL)	Hemoglobin<9.7				
	9.7≤Hemoglobin<10.6	0.58 [0.24, 1.39]	0.220		
	Hemoglobin≥10.6	0.39 [0.16, 0.92]	0.032		
Calcium (mg/dL)	Calcium<7.9				
	7.9≤Calcium<8.5	1.05 [0.45, 2.45]	0.910		
	Calcium≥8.5	0.60 [0.24, 1.47]	0.260		
Phosphorus (mg/dL)	Phosphorus<4.1				
	4.1≤Phosphorus<5.0	0.34 [0.15, 0.77]	0.010		
	Phosphorus≥5.0	0.12 [0.03, 0.39]	0.001		
Albumin (g/dL)	Albumin<3.3				
	3.3≤Albumin<3.6	0.20 [0.07, 0.58]	0.003	0.20 [0.06, 0.71]	0.013^a^
	Albumin≥3.6	0.10 [0.03, 0.31]	<0.001	0.12 [0.03, 0.42]	0.001^a^
Potassium (mEq/L)	Potassium<4.2				
	4.2≤Potassium<4.7	0.47 [0.20, 1.13]	0.091	0.75 [0.31, 1.79]	0.520^a^
	Potassium≥4.7	0.34 [0.11, 1.00]	0.051	0.48 [0.15, 1.49]	0.210^a^
24-h urine volume (mL/day)	24-h urine volume<500				
	500≤24-h urine volume<1000	0.45 [0.18, 1.11]	0.083	0.40 [0.17, 0.96]	0.039^b^
	24-h urine volume≥1000	0.54 [0.22, 1.32]	0.170	0.50 [0.18, 1.11]	0.280^b^
mCCI	Low (0–5)				
	Moderate (6–7)	1.60 [0.73, 3.47]	0.240		
	High (8–15)	0.68 [0.22, 2.09]	0.500		
SGA	Malnourished (1–5)				
	Nourished (6–7)	0.40 [0.18, 0.89]	0.025		
Diabetes	Present (vs. absent)	3.07 [1.31, 7.18]	0.010	2.73 [1.13, 6.57]	0.025^b^
Peritonitis	Present (vs. absent)	1.58 [0.78, 3.19]	0.210		
Hospitalization	Present (vs. absent)	1.70 [0.71, 4.08]	0.230		
**Technical failure**					
Age	Elderly PD (vs. younger PD)	1.46 [0.87, 2.45]	0.160	1.43 [0.81, 2.53]	0.220^c^
Sex	Female (vs. male)	0.90 [0.59, 1.38]	0.630		
Hemoglobin (g/dL)	Hemoglobin<9.7	1 (reference)			
	9.7≤Hemoglobin<10.6	0.87 [0.49, 1.55]	0.650		
	Hemoglobin≥10.6	0.98 [0.60, 1.60]	0.930		
Calcium (mg/dL)	Calcium<7.9	1 (reference)			
	7.9≤Calcium<8.5	1.16 [0.68, 1.99]	0.580		
	Calcium≥8.5	1.13 [0.69, 1.83]	0.630		
Phosphorus (mg/dL)	Phosphorus<4.1	1 (reference)			
	4.1≤Phosphorus<5.0	1.13 [0.67, 1.90]	0.660		
	Phosphorus≥5.0	1.00 [0.60, 1.68]	0.990		
Albumin (g/dL)	Albumin<3.3	1 (reference)			
	3.3≤Albumin<3.6	0.79 [0.46, 1.36]	0.400		
	Albumin≥3.6	0.68 [0.42, 1.09]	0.110		
Potassium (mEq/L)	Potassium<4.2	1 (reference)		1 (reference)	
	4.2≤Potassium<4.7	0.82 [0.50, 1.33]	0.420	0.80 [0.49, 1.30]	0.360^c^
	Potassium≥4.7	1.16 [0.70, 1.91]	0.570	0.99 [0.58, 1.69]	0.970^c^
24-h urine volume (mL/day)	24-h urine volume<500	1 (reference)		1 (reference)	
	500≤24-h urine volume<1000	0.99 [0.58, 1.67]	0.960	0.87 [0.50, 1.52]	0.620^c^
	24-h urine volume≥1000	1.47 [0.86, 2.50]	0.160	1.35 [0.78, 2.33]	0.280^c^
mCCI	Low (0–5)	1 (reference)			
	Moderate (6–7)	1.34 [0.84, 2.15]	0.220		
	High (8–15)	1.15 [0.67, 1.96]	0.620		
SGA	Malnourished (1–5)	1 (reference)			
	Nourished (6–7)	0.78 [0.50, 1.23]	0.290		
Diabetes	Present (vs. absent)	1.40 [0.92, 2.11]	0.110	1.13 [0.73, 1.76]	0.590^c^
Peritonitis	Present (vs. absent)	1.87 [1.23, 2.84]	0.003	1.79 [1.15, 2.80]	0.010^c^
Hospitalization	Present (vs. absent)	2.16 [1.24, 3.79]	0.007		

*Variables that were adopted in the final stepwise-multivariate model alone were presented.

^a^ Adjusted for age, hemoglobin, phosphorus, albumin, potassium, 24-h urine volume, SGA, and diabetes.

^b^ Adjusted for age, 24-h urine volume, SGA, and diabetes.

^c^ Adjusted for age, albumin, 24-h urine volume, diabetes, and peritonitis.

Abbreviations: SHR, sub-hazard ratios; CI, confidence interval; PD, peritoneal dialysis; SGA, subjective global assessment.

When differences in patient death rates between elderly PD group and elderly HD groups were compared using cumulative incidence function test, the distribution between the PD group and HD group did not differ significantly (Fig 2C, P = 0.987). After adjusting for group (elderly PD vs. elderly HD), age, hemoglobin, albumin, 24-h urine volume, SGA, diabetes, and hospitalization, the risk of death 0.73 times lower in the elderly PD group than in the elderly HD group, but the difference was not statistically significant (Table 2, P = 0.380).

Common causes of technical failure in elderly PD patients included peritonitis, patient preference, pleural effusion, and abdominal surgeries (Table 3). The incidence of peritonitis was significantly higher in elderly PD patients than those of younger PD patients (P<0.001). For microorganism of the 1^st^ episode of peritonitis, 118 events occurred in all subjects and the difference of microorganism across the age groups was not significant (S2 Table).

**Table 3:** Reasons for technical failure according to age groups among peritoneal dialysis patients.

Reasons for technical failure	PD, ≤49 y	PD, 50~64 y	PD, ≥65 y
**No. of patients**	**205**	**192**	**95**
**Overall technical failure rate**	**36 (17.5)**	**36 (18.7)**	**20 (21.1)**
Peritonitis	8 (22)	12(33)	6 (30)
Patient preference	4 (11)	5 (14)	3 (15)
Inadequate fluid ultrafiltration or solute clearance	3 (11)	1 (3)	2 (10)
Pleural effusion	2 (6)	1 (3)	2 (10)
Unable to manage self-care	0 (0)	1 (3)	2 (10)
Planned transfer	8 (22)	4 (11)	1 (5)
Tunnel exit site infection	1 (3)	0 (0)	1 (5)
Dialysate leak	1 (3)	1 (3)	1 (3)
Hernia	1 (3)	1 (3)	1 (3)
Abdominal surgery	1 (3)	1 (3)	1 (3)
Unknown	7 (20)	8 (22)	9 (27)

Abbreviations: PD, peritoneal dialysis.

### Quality of Life and Depression Scores of Elderly PD Patients Compared to Younger Patients and Elderly HD Patients

The KDQOL-36 and BDI status were assessed in a subset of patients. Among the 3 PD groups, the baseline (3-month) values were similar (Fig A in S1 File). In the symptom, effect, and burden domains, the elderly PD group showed significant improvements in the 12-month scores, as compared with the ≤49-year PD group (P = 0.03, P = 0.004, and P = 0.003, respectively; Fig B in S1 File). Excluding the physical component scores, no significant differences between the baseline and 12-month KDQOL-36 scores were observed between the PD groups.

Despite the poorest baseline BDI in elderly PD patients, this difference disappeared after 12 months (Fig C in S1 File), and its improvement was significant, as compared to that in the ≤49-year group (12-month change of BDI: 7.1 ± 12.1 *vs*. -0.3 ± 7.8; P = 0.003, Fig D in S1 File).

Compared with elderly HD patients, the baseline value of KDQOL-36 in elderly PD patients was similar (Fig E in S1 File). However, the 12-month changes in BDI of elderly PD patients were significantly improved in the effects and burden domains (P = 0.030, P = 0.004; Fig F in S1 File), whereas the physical score was significantly lower (P = 0.001; Fig F in S1 File).

While the elderly PD group showed a higher baseline BDI score, as compared to the HD group (20.3 ± 12.0 *vs*. 16.4 ± 10.4; P = 0.040, Fig G in S1 File), the 12-month changes were similar between the 2 groups (Fig H in S1 File).

### Risk Factors of Patient or Technical Survival among PD Patients or Elderly Patients

Age, hemoglobin, phosphorus, albumin, potassium, 24-h urine volume, SGA, and diabetes were variables with P<0.2 in the univariate analysis of patient survival among PD patients. Interaction was observed between albumin and diabetes and between phosphorus and albumin. The final model included age (younger PD (reference) *vs*. elderly PD: sub-hazard ratios (SHR) [95% CI], 2.99 [1.32, 6.76], P = 0.009) and albumin (albumin<3.3 g/dL (reference) *vs*. albumin≥3.6 g/dL: SHR [95% CI], 0.12 [0.03, 0.42], P = 0.001) as factors with a significance level of 0.05 in the stepwise (entry level 0.05, stay level 0.05) multivariate analysis of patient death. We additionally analyzed the model without laboratory data. In the stepwise model adjusted for age, 24-h urine volume, SGA, and diabetes, diabetes (SHR [95% CI], 2.73 [1.13, 6.57], P = 0.025) and 24-h urine volumes (24-h urine volume<500 mL/day (reference) vs. 500≤24-h urine volume<1000 mL/day: SHR [95% CI], 0.40 [0.17, 0.96], P = 0.039) were significant risk factors of patient death in the 2 PD groups (Table 2).

Next, in the univariate analysis of technical survival among PD patients, age, albumin, 24-h urine volume, diabetes and peritonitis were used as correction parameters in the multivariate analysis. The final model included peritonitis (SHR [95% CI], 1.79 [1.15, 2.80], P = 0.010) as a significant factor (Table 2).

The univariate analysis of patient survival among elderly patients to examine factors affecting mortality showed that age, hemoglobin, albumin, 24-h urine volume, SGA, diabetes, and hospitalization significantly affected mortality at P<0.2; these were included in the multivariate analysis using a stepwise method. Albumin (albumin<3.3 g/dL (reference) *vs*. albumin≥3.6 g/dL: SHR [95% CI], 0.36 [0.18, 0.69], P = 0.002) and hospitalization (SHR [95% CI], 3.20 [1.35, 7.54], P = 0.008) were selected as factors affecting elderly patient mortality (Table 4).

**Table 4:** Independent risk factors of patient death in elderly patients.

Patient death		Univariate competing risk regression		Multivariate competing risk regression*	
Variable	Category	SHR [95% CI]	P-value	SHR [95% CI]	P-value ^a^
Group	Elderly PD (vs. elderly HD)	1.03 [0.57, 1.86]	0.920	0.73[0.37, 1.47]	0.380
Age	Age≥70 (vs. age<70)	1.56 [0.85, 2.80]	0.150		
Sex	Female (vs. male)	0.86 [0.48, 1.54]	0.620		
Hemoglobin (g/dL)	Hemoglobin<9.7	1 (reference)			
	9.7≤Hemoglobin<10.6	0.56 [0.28, 1.10]	0.093		
	Hemoglobin≥10.6	0.46 [0.23, 0.88]	0.020		
Calcium (mg/dL)	Calcium<7.9	1 (reference)			
	7.9≤Calcium<8.5	1.14 [0.59, 2.19]	0.690		
	Calcium≥8.5	0.73 [0.35, 1.51]	0.400		
Phosphorus (mg/dL)	Phosphorus<4.1	1 (reference)			
	4.1≤Phosphorus<5.0	0.75 [0.40, 1.40]	0.370		
	Phosphorus≥5.0	0.78 [0.38, 1.61]	0.500		
Albumin (g/dL)	Albumin<3.3	1 (reference)			
	3.3≤Albumin<3.6	0.39 [0.17, 0.88]	0.023	0.34 [0.13, 0.87]	0.024
	Albumin≥3.6	0.35 [0.19, 0.64]	0.001	0.36 [0.18, 0.69]	0.002
Potassium (mEq/L)	Potassium<4.2	1 (reference)			
	4.2≤Potassium<4.7	1.06 [0.54, 2.08]	0.870	1.49 [0.72, 3.12]	0.280
	Potassium≥4.7	1.13 [0.59, 2.18]	0.720	2.00 [0.99, 4.00]	0.051
24-h urine volume (mL/day)	24-h urine volume<500	1 (reference)			
	500≤24-h urine volume<1000	0.86 [0.46, 1.63]	0.650	0.88 [0.47, 1.64]	0.690
	24-h urine volume≥1000	0.54 [0.24, 1.28]	0.160	0.55 [0.23, 1.33]	0.190
mCCI	Low (0–5)	1 (reference)			
	Moderate (6–7)	1.08 [0.60, 1.97]	0.790		
	High (8–15)	0.92 [0.44, 1.98]	0.850		
SGA	Malnourished (1–5)	1 (reference)			
	Nourished (6–7)	0.66 [0.38, 1.17]	0.160		
Diabetes	Present (vs. absent)	1.98 [1.05, 3.75]	0.035		
RRT related event	Present (vs. absent)	1.28 [0.72, 2.26]	0.400		
Hospitalization	Present (vs. absent)	3.07 [1.45, 6.52]	0.004	3.20 [1.35, 7.54]	0.008

*Variables that were adopted in the final stepwise-multivariate model alone were presented.

^a^ Adjusted for group (elderly PD vs. elderly HD), age, hemoglobin, albumin, 24-h urine volume, SGA, diabetes, and hospitalization.

Abbreviations: SHR, sub-hazard ratios; CI, confidence interval; PD, peritoneal dialysis; HD, hemodialysis; SGA, subjective global assessment; RRT, renal replacement therapy.

## Discussion

The present nationwide multi-center prospective cohort study investigated the clinical outcomes of elderly PD patients, as compared with younger PD and elderly HD patients. The survival rate of elderly PD patients was inferior to those of younger patients. However the technical survival rate was not different among the younger PD groups. On the other hand, the patient survival rate of elderly PD did not differ significantly from that of elderly HD patients. In addition, elderly PD subjects showed significant improvement in 1-year BDI scores, as compared to younger PD patients. Significant risk factors for the PD patient survival included age and low albumin levels, for the technical survival, high peritonitis rate. Low albumin level and high hospitalization were significant risk factors for patient survival of elderly patients.

The elderly PD patients had more comorbidity, poorer performance, and were more frequently malnourished including low albumin and phosphorus levels; their survival rates were inferior to those of younger PD patients. However, the technical survival rate was similar between elderly and younger PD patients. Regardless of age, peritonitis was the most common cause of technical failure in PD patients. The incidence of peritonitis of elderly PD patients was higher than that of younger PD patients, but microbiology was similar between the 2 groups.

De Vecchi et al. likewise reported that patient survival was poorer and the incidence of peritonitis was higher in the elderly than younger PD patients, whereas the technical survival was similar [35]. Lim et al. also recently reported that the hazard ratios for technical failure were similar across the age groups despite higher risk of peritonitis-related mortality [36]. Yang et al. reported inferior patient survival in elderly PD than in younger PD patients, but similar technical survival [37]; and additional studies similarly showed no differences in the technical survival rates between elderly and younger PD patients [22, 38]. In the present study, patient and technical survival rate was analyzed using a competing risk model, up-to-date statistical technique.

In the present study, the patient survival rate did not differ significantly between elderly PD and HD patients. Conversely, Winkelmayer et al. reported that the death rate of elderly PD patients was higher than that of elderly HD patients in the US [39]. We speculate that this discrepancy is due to the relatively rare prescription of PD in the US. Furthermore, differences in ethnicity or the timing of the studies may have a role. Recently, Lee et al. also reported that the patient survival rate of elderly PD patients was worse than that of elderly HD patients using data from the Korean Health Insurance Review and Assessment Service [40]. The reason for this discrepancy might be the use of a manipulative definition of dialysis rather than real “end-stage renal failure”; and, accordingly, patients with acute kidney injury might have been included in the HD group. Additionally, the relatively small number of patients in the present study should also be considered.

Next, we investigated the QOL of elderly PD patients. Harris et al. reported no significant difference in QOL according to modality in elderly patients in their 1-year prospective study [38]; and our data revealed that the baseline and 1-year changes in KDQOL-36 scores did not significantly differ between groups. However, in Harris’ study, the QOL score of elderly PD remained the same or was reduced during the follow-up period, whereas in our study, all domain scores, except for the physical domain, were improved. Moreover, in the current study, the improvements in the KDQOL-36 scores of the elderly PD patients tended to be superior to that of younger PD and elderly HD patients.

Importantly, elderly PD patients showed more improvement in BDI than younger PD patients. The elderly PD group was more dependent on social and familial supports than the younger groups, and they had a lower education level. Thus, it was notable for them to show higher BDI improvements. QOL is recently being emphasized as an important treatment goal; considering that QOL and depression are associated with patient morbidity and mortality [41, 42], further large-scale studies are necessary to confirm this finding.

The study data indicated that albumin levels are important to mortality of PD and elderly patients i.e., mortality rates were high in PD patients with low albumin levels. Low albumin was also an independent risk factor for survival in elderly patients, due to its reflection of nutritional status or inflammation [43]. In fact, Kang et al. suggested that low albumin is associated with mortality in PD patients [44]. Similarly, Yang et al. reported that low serum cholesterol and albumin are significant risk factors for both patient and technical survival in PD patients [37]. Joly D et al. reported that nutritional status is important risk factors of mortality in elderly patients [45]. Since low phosphorus, low albumin, low potassium, and poor SGA in PD patients, and low hemoglobin and low albumin in elderly patients were significant univariate risk factors of patient death in the present study, we concluded that nutritional status of ESRD patient is associated with patient survival.

In our study, some traditional risk factors of patient death such as diabetes, residual renal function (24-h urine volume), and hypokalemia were not significant in the multivariate model with laboratory findings. Because of the strong interaction of diabetes and albumin, we re-analyzed our data without laboratory findings and found that diabetes and low residual renal function were significant risk factors for death of PD patients. Hypokalemia is a marker of malnutrition, similar to hypoalbuminemia [46, 47]; hence, because of clinical correlation with each other, it became insignificant in this analysis. In addition, the relative small sample size of elderly PD could be one of the causes of statistical non-significance.

Considering the benefits of home-based dialysis, by enhancing the PD success rate, PD may be more frequently requested in the elderly. However, other modalities should be recommended in elderly patients with several risk factors such as hypoalbuminemia and high hospitalization rate. Moreover, peritonitis was the most common cause of technical failure; therefore, reducing the incidence of peritonitis is important to ensure the success of PD in elderly patients.

The main limitation of our study was the relatively small sample size of elderly PD patients; a large-scale, long-term, prospective study is needed in the future to confirm our findings. Additionally, because the modality of dialysis is influenced by medical (e.g. comorbidities) and non-medical factors (e.g. social or familial support, occupation), the choice of HD or PD has inherent differences. Therefore, selection bias was inevitable in the choice of dialysis modality in elderly patients.

Overall, our results suggested that PD is a comparable modality to manage elderly ESRD patients, considering the psychological and its comparable outcomes to HD. In order to enhance the success rate of PD, judicious screening of patients considering such as nutritional status or hospitalization rate and constant efforts to reduce the incidence of peritonitis are required.

## Supporting Information

S1 File Quality of life (QOL) and depression scores of elderly peritoneal dialysis (PD) patients compared with younger PD patients and elderly hemodialysis (HD) patients. Baseline KDQOL-36 scores of the 3 PD groups (Fig A). Twelve-month changes of KDQOL-36 scores of the 3 PD groups (Fig B). Baseline BDI of the 3 PD groups (Fig C). Twelve-month changes of BDI of the 3 PD groups (Fig D). Baseline KDQOL-36 scores of the 2 elderly groups (Fig E). Twelve-month changes of KDQOL-36 scores of the elderly 2 groups (Fig F). Baseline BDI of the 2 elderly groups (Fig G). Twelve-month changes of BDI of the 2 elderly groups (Fig H). KDQOL-36, Kidney Disease Quality of Life-36; BDI, Beck’s Depression Inventory. Repeated measure ANOVA or Student’s t-test was used as appropriate.
(TIF)

S1 Table Cause of death according to age and dialysis modality. (DOCX)

S2 Table Incidence and description of microorganism of peritonitis according to age groups. (DOCX)
