# Supplementary figures and images for: Elderly Peritoneal Dialysis Compared with Elderly Hemodialysis Patients and Younger Peritoneal Dialysis Patients: Competing Risk Analysis of a Korean Prospective Cohort Study
Source: PLoS One. 2015 Jun 29;10(6):e0131393. doi: 10.1371/journal.pone.0131393 (PMC4488000; doi:10.1371/journal.pone.0131393)

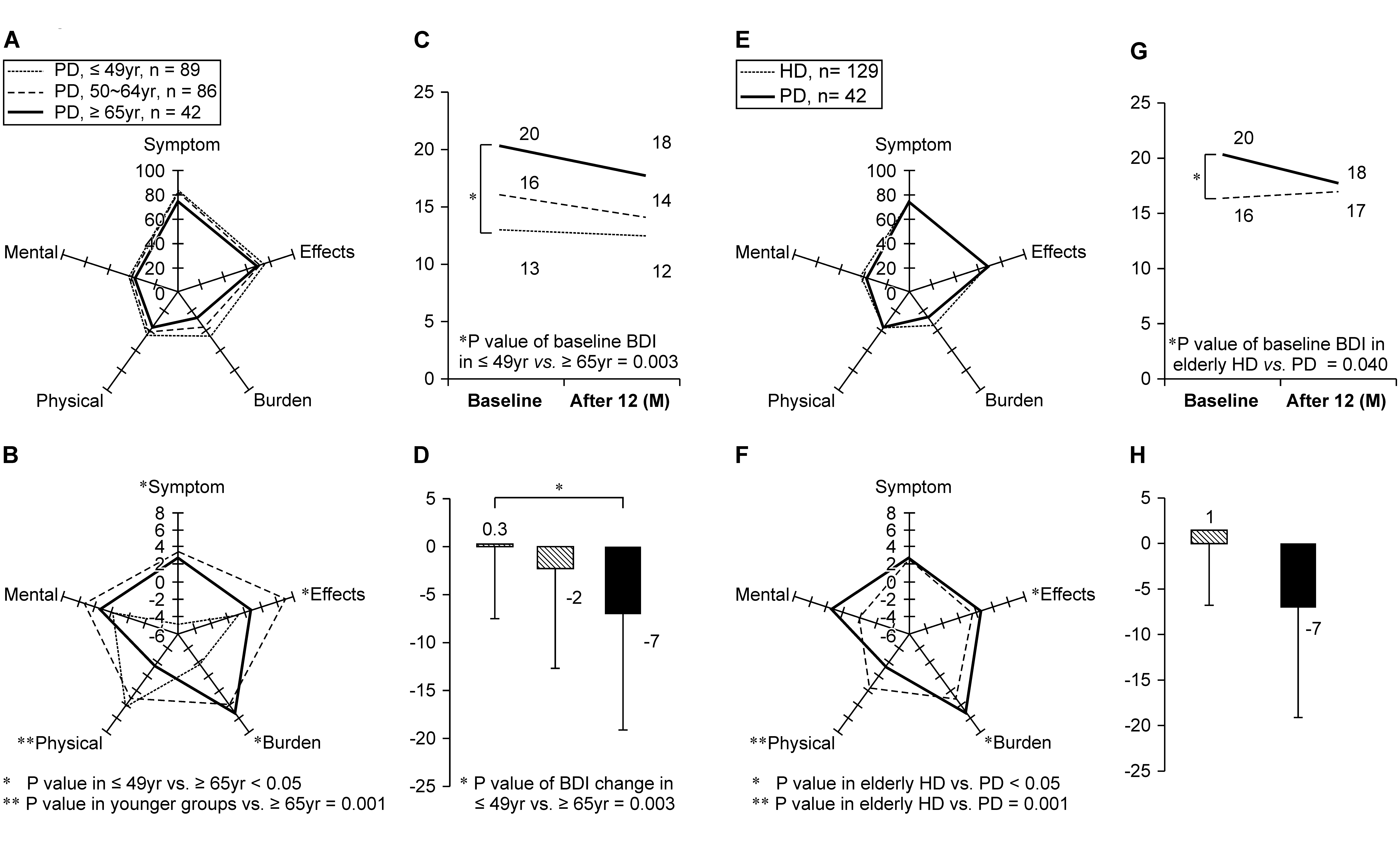

Supplement: S1 File — Baseline KDQOL-36 scores of the 3 PD groups (Fig A). Twelve-month changes of KDQOL-36 scores of the 3 PD groups (Fig B). Baseline BDI of the 3 PD groups (Fig C). Twelve-month changes of BDI of the 3 PD groups (Fig D). Baseline KDQOL-36 scores of the 2 elderly groups (Fig E). Twelve-month changes of KDQOL-36 scores of the elderly 2 groups (Fig F). Baseline BDI of the 2 elderly groups (Fig G). Twelve-month changes of BDI of the 2 elderly groups (Fig H). KDQOL-36, Kidney Disease Quality of Life-36; BDI, Beck’s Depression Inventory. Repeated measure ANOVA or Student’s t-test was used as appropriate. (TIF) [file pone.0131393.s001.tif]
