# Supplementary material for: Elderly Peritoneal Dialysis Compared with Elderly Hemodialysis Patients and Younger Peritoneal Dialysis Patients: Competing Risk Analysis of a Korean Prospective Cohort Study
Source: PLoS One. 2015 Jun 29;10(6):e0131393. doi: 10.1371/journal.pone.0131393 (PMC4488000; doi:10.1371/journal.pone.0131393)
Supplement: S1 Table — (DOCX) [file pone.0131393.s002.docx]

**Table S1. Cause of death according to age and dialysis modality**

| Death (n=82) | | PD, ≤49 y | PD, 50~64 y | PD, ≥65 y | HD, ≥ 65 y |
| --- | --- | --- | --- | --- | --- |
| Patients (n) | | 205 | 192 | 95 | 315 |
| All cause of death, n (%) | | 5 (2.4) | 16 (8.3) | 22 (23.2) | 39 (12.4) |
| Cardiovascular cause, n (%) | | 1 (0.4) | 5 (2.6) | 7 (7.4) | 13 (4.1) |
| Infectious cause, n (%) | | 2 (1.0) | 5 (2.6) | 6 (6.3) | 8 (2.6) |
| Other cause, n (%) | | 2(1.0) | 6 (3.1) | 9 (9.5) | 18 (5.7) |
|  | Hyperkalemia | 0 | 0 | 0 | 1 |
|  | Hypoglycemia | 1 | 0 | 0 | 0 |
|  | Malignant disease | 0 | 1 | 0 | 3 |
|  | Cachexia | 0 | 0 | 1 | 0 |
|  | Accident related to treatment | 0 | 0 | 1 | 0 |
|  | Unspecified | 1 | 2 | 2 | 8 |
|  | Unknown | 0 | 3 | 5 | 6 |

Abbreviations: PD, peritoneal dialysis; HD, hemodialysis
