# Supplementary material for: Elderly Peritoneal Dialysis Compared with Elderly Hemodialysis Patients and Younger Peritoneal Dialysis Patients: Competing Risk Analysis of a Korean Prospective Cohort Study
Source: PLoS One. 2015 Jun 29;10(6):e0131393. doi: 10.1371/journal.pone.0131393 (PMC4488000; doi:10.1371/journal.pone.0131393)
Supplement: S2 Table — (DOCX) [file pone.0131393.s003.docx]

**Table S2. Incidence and description of microorganism of peritonitis according to age groups**

| Microorganism | | PD, ≤49 y | PD, 50~64 y | PD, ≥65 y | P |
| --- | --- | --- | --- | --- | --- |
| Patients (n) | | 205 | 192 | 95 |  |
| Incidence of peritonitis, n (%) | | 42 (20.5) | 42 (21.9) | 34 (35.8) | <0.001 |
| Total culture, n (%) | | 34 (100) | 34 (100) | 50 (100) |  |
| Gram (+) bacteria | | 9 (26.5) | 11 (32.3) | 21 (42) | 0.172 |
|  | Staphylococcus aureus | 3 | 4 | 8 |  |
|  | Coagulase negative Staphylococcus | 1 | 1 | 3 |  |
|  | Streptococcus | 0 | 5 | 5 |  |
|  | Enterococcus (Strep. faecalis/faecium) | 1 | 1 | 2 |  |
|  | Diphtheroids (Corynebacteria) | 0 | 0 | 1 |  |
|  | Gram (+) organism, other | 4 | 0 | 2 |  |
| Gram (-) bacteria | | 9 (26.5) | 11 (32.3) | 11 (22) | 0.978 |
|  | Pseudomonas | 2 | 2 | 0 |  |
|  | E. coli, Klebsiella or Serratia | 1 | 3 | 5 |  |
|  | Enterobacter species | 0 | 1 | 2 |  |
|  | Roseomonas gilardii | 5 | 1 | 2 |  |
|  | Gram (-) organisms, other | 1 | 4 | 2 |  |
| Anaerobic bacteria | | 0 (0) | 1 (3) | 0 (0) |  |
| Culture negative | | 16 (47) | 11 (32.4) | 18 (36) |  |

Abbreviations: PD, peritoneal dialysis
